# Universal‐Bac^3^Gel: A 3D Biofilm‐Relevant Matrix That Supports In Vitro Growth and Biofilm Formation of ESKAPE Pathogens

**DOI:** 10.1002/mbo3.70371

**Published:** 2026-08-12

**Authors:** Emanuela Peluso, Sebastião van Uden, Sonja Visentin, Paola Petrini, Daniela Peneda Pacheco, Livia Visai

**Affiliations:** ^1^ Molecular Medicine Department (DMM), Centre for Health Technologies (CHT), Unità di Ricerca (UdR) INSTM, Operative Unit (OU) of the Interuniversity Center for the Promotion of the 3Rs Principles in Teaching and Research (Centro 3R) University of Pavia Pavia Italy; ^2^ Bac^3^Gel® Lda, Edifício Inovação II Porto Salvo Portugal; ^3^ Department of Molecular Biotechnology and Health Sciences University of Torino Turin Italy; ^4^ BioAvatar Lab, Department of Chemistry Materials, and Chemical Engineering “G. Natta”, Politecnico di Milano Milan Italy; ^5^ Department of Prevention and Rehabilitation in Occupational Medicine and Speciality Medicine, UOR6 Nanotechnology Laboratory Istituti Clinici Scientifici Maugeri IRCCS Pavia Italy

**Keywords:** 3D cultures, antimicrobial resistance, biofilm‐relevant environment, ESKAPE pathogens, gradients, microbial ecology, translational model

## Abstract

Human microbiota is increasingly considered to shape health and disease, drawing interest of pharma and biotech industries in advanced models of in vitro human microbiome to streamline drug development. In this context, Universal‐Bac^3^Gel represents a new generation of 3D biomaterials designed to mimic the properties of human mucus and biofilm features, including micro‐gradients that replicate the heterogeneous environments colonized by microorganisms in the human body. To evaluate the suitability of Universal‐Bac^3^Gel for studying clinically relevant species in antimicrobial resistance, the so‐called ESKAPE pathogens (*Enterococcus faecium, Staphylococcus aureus, Klebsiella pneumoniae, Acinetobacter baumannii, Pseudomonas aeruginosa, and Enterobacter cloacae*) were cultured within this 3D environment. Bacterial growth was monitored at 24‐ and 48‐h post‐inoculation via spot plating, while viability, spatial distribution, and organization were assessed by confocal laser scanning microscopy. All ESKAPE strains successfully grew throughout the structure of Universal‐Bac^3^Gel. Distinct 3D biofilm architectures were observed across species, ranging from diffuse colonization to compact microcolony formation, in agreement with species‐specific biofilm patterns. Ciprofloxacin susceptibility assays revealed reduced susceptibility of bacteria cultured within Universal‐Bac^3^Gel compared with their planktonic counterparts, supporting the development of biofilm‐associated tolerance phenotypes. Consistent with these findings, crystal violet staining confirmed the accumulation of biofilm‐associated biomass within the hydrogel. Notably, the platform's ready‐to‐use 96‐well format allowed direct comparison of these high‐priority pathogens under standardized conditions, highlighting species‐specific biofilm traits that would be difficult to discern in conventional two‐dimensional culture systems. This work highlights the versatility of Universal‐Bac^3^Gel as a biofilm‐relevant in vitro platform for studying pathogen colonization, biofilm development and antimicrobial susceptibility under controlled conditions.

## Graphical abstract

Universal‐Bac^3^Gel, a 3D biofilm‐relevant matrix with oxygen and nutrient gradients, supports the growth and species‐specific biofilm architectures of all ESKAPE pathogens, offering a physiologically relevant platform for antimicrobial testing and microbiological research.

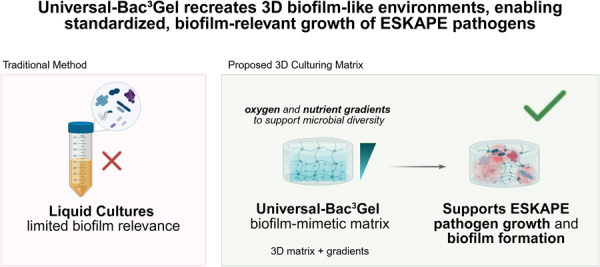

## 1 Introduction

The human resident bacterial flora, i.e., all microbial populations that establish a symbiotic relationship with the tissues of the human organism, is collectively known as the microbiota. Current quantitative analyses indicate that the total number of microbial and human cells in the body is approximately equal, with a near 1:1 ratio, although this estimate varies depending on host‐specific factors such as body size, diet, and microbiome composition (Sender et al. 2016). While all human organisms are anatomically similar, multiple factors influence the development of bacterial populations, determining the specificity of the microbiota for each individual.

In natural and host‐associated environments, bacteria predominantly exist as biofilms, structured microbial communities embedded within a self‐produced extracellular matrix that provides protection from environmental stresses, host immune responses, and antimicrobial agents. It is estimated that roughly 99% of bacteria on Earth exist in biofilms, while only about 1% persist in a free‐floating, planktonic state (Flemming and Wuertz 2019). Nevertheless, most in vitro studies still rely on planktonic cultures, which fail to reproduce the complex structural and physiological characteristics of bacteria growing in vivo.

To address these limitations, innovative cultivation strategies have emerged over the past decade, including growth in natural habitats and the use of modified media that enable a broader recovery of microbial diversity. However, conventional two‐dimensional (2D) models for studying bacterial infections remain limited in their ability to accurately reproduce the in vivo environment, as they lack pathogen‐specific cell types, intercellular signaling, and biomechanical cues (Fasciano et al. 2019); (Bermudez‐Brito et al. 2013; Shi et al. 2019).

These shortcomings become particularly critical when investigating infections at mucosal surfaces, where the biochemical and physical microenvironment plays a key regulatory role. Mucosal surfaces constitute the primary ecological niche for microbial colonization and infection. The human mucosa is coated with mucus layers composed of mucins, lipids, and other proteins such as immunoglobulins, which serve both as a physical barrier and as a dynamic biochemical habitat for bacteria (Cone 2009). The viscoelastic and heterogeneous nature of mucus creates spatial gradients of oxygen, nutrients, and antimicrobial peptides that strongly influence microbial behavior and biofilm development (Lieleg and Ribbeck 2011). These features are rarely reproduced in traditional in vitro systems, underscoring the need for advanced models that more faithfully mimic the biophysical properties of mucus, as well as the unified mucus and biofilm niche— mucofilm (Mortensen et al. 2025).

Recent technological advances highlight the promise of three‐dimensional (3D) organotypic systems and organ‐on‐a‐chip platforms that replicate physiological and pathological tissue functions with greater fidelity, providing improved tools to study host‐microbe interactions and infection dynamics (Rikken et al. 2023; Nasiri et al. 2024). At the same time, the escalating global challenge of antimicrobial resistance highlights the importance of physiologically relevant systems to investigate pathogen adaptation, persistence, and treatment response (Santos and Coelho 2025; Higazy et al. 2024).

**Figure 1:**
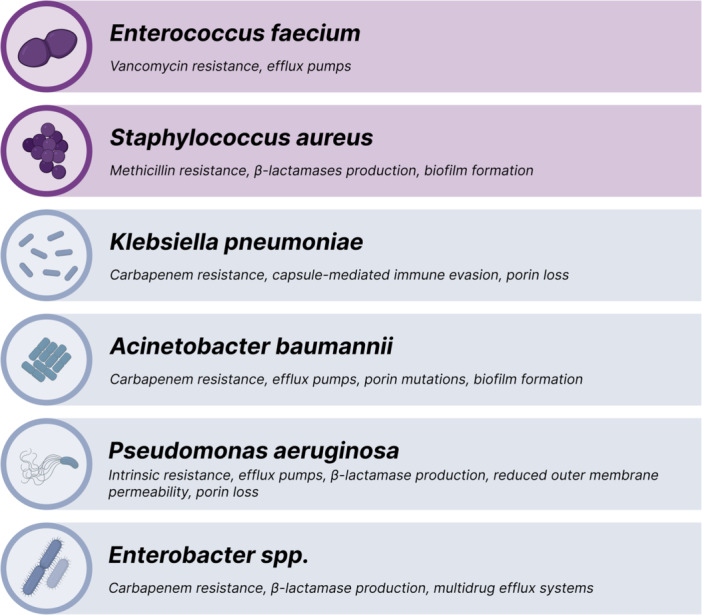
Overview of the ESKAPE pathogens, divided into Gram‐positive (purple) and Gram‐negative (blue) groups, and their major antimicrobial resistance mechanisms. The ESKAPE group comprises six clinically significant species ‐Enterococcus faecium (Santajit and Indrawattana 2016) (Munita and Arias 2016); Staphylococcus aureus (Pendleton et al. 2013) (Munita and Arias 2016) Klebsiella pneumoniae (Pendleton et al. 2013) (Santajit and Indrawattana 2016); Acinetobacter baumannii (Pendleton et al. 2013) (Santajit and Indrawattana 2016), Pseudomonas aeruginosa (Munita and Arias 2016) (Boucher et al. 2009) and Enterobacter spp. (Santajit and Indrawattana 2016). Each exhibits distinct resistance strategies, including β‐lactamase production, efflux pumps, porin modifications, and biofilm formation.

In particular, surveillance data have identified a group of nosocomial bacteria—collectively known as the ESKAPE pathogens—that are responsible for the majority of hospital‐acquired infections worldwide. This group includes both Gram‐positive (Figure 1 purple) and Gram‐negative species (Figure 1 blue), notorious for multidrug resistance and their ability to “escape” the effects of antibiotics (Santajit and Indrawattana 2016; Bush and Jacoby 2010). These organisms represent leading causes of severe infections, particularly among critically ill or immunocompromised patients (Ram et al. 2010).

In this context, Universal‐Bac^3^Gel represents a three‐dimensional biomaterial designed to mimic key structural, biochemical, and rheological features of mucus and biofilm environments. The hydrogel reproduces physiologically relevant characteristics, including diffusion limitations, oxygen and nutrient gradients, and viscoelastic behavior (storage modulus G′ > G″), creating conditions that support microbial colonization and spatial organization (Pacheco et al. 2023). Several in vitro and ex vivo systems have been developed to model mucus‐associated infections and biofilm formation, including artificial sputum medium (ASM), AirGels, and organotypic tissue co‐cultures (Crabbé et al. 2019; Harrison and Diggle 2016; Möckel et al. 2022; Phogat et al. 2023). While these models have substantially improved our ability to investigate host‐microbe interactions and biofilm physiology, there remains a need for scalable and reproducible platforms that can accommodate a broad range of clinically relevant pathogens.

Universal‐Bac^3^Gel is provided as a ready‐to‐use matrix compatible with standard 96‐well plate formats, facilitating experimental reproducibility and high‐throughput workflows. The physicochemical properties of the hydrogel provide a more physiologically relevant environment than conventional planktonic cultures. Such models are particularly relevant for studying ESKAPE pathogens, whose persistence and therapeutic recalcitrance are frequently associated with biofilm formation and altered antimicrobial susceptibility.

The aim of this study was therefore to evaluate the ability of Universal‐Bac^3^Gel to support the growth, viability, and three‐dimensional organization of all six ESKAPE pathogens (reference strains). In addition, we investigated ciprofloxacin susceptibility under both planktonic and Universal‐Bac^3^Gel culture conditions and assessed biofilm‐associated biomass accumulation by crystal violet staining. Together, these analyses provide structural and functional evidence supporting the use of Universal‐Bac^3^Gel as a biofilm‐relevant in vitro platform for the study of clinically important bacterial pathogens.

## 2 Materials and Methods

### 2.1 Bac^3^Gel Technology

By replicating key properties of the biofilm environment, Bac^3^Gel (https://www.bac3gel.com/) provides an advanced 3D material exhibiting micro‐gradients for the culture of microorganisms. In this study, Universal‐Bac^3^Gel was exploited as a general‐use 3D support for bacteria colonization and growth, which is provided in a standard 96‐well flat‐bottomed sterile microplate, as ready to use. Universal‐Bac^3^Gel composition is formulated to reproduce the biofilm stratified environment, creating a diffusion‐limited environment with oxygen and nutrient gradients (Pacheco et al. 2023; Vargas et al. 2025). A detailed step‐by‐step protocol for culturing single strains in Universal‐Bac^3^Gel is available in Appendix 1 (Link A1).

### 2.2 Rheological Characterization

The viscoelastic properties of Universal‐Bac^3^Gel produced in different media (7.07 mg mL^−1^ NaCl, MHB, and LB) were evaluated using an Anton Paar MCR501 Rheometer (Austria) with a 25 mm diameter plate geometry (serial number 52530/19910) at 25°C. The linear viscoelastic region (LVR) was determined through strain sweep analyses employing a logarithmic ramp strain varying from 0.1% to 1000% at a frequency of 1 Hz. Oscillatory frequency sweeps were further performed to evaluate both storage, *G’*, and dissipative, *G″*, moduli, at 0.5% (at strain amplitudes within the linear regime) with frequencies changing logarithmically in the 0.1–20 Hz range.

### 2.3 Oxygen Tension Measurements

O_2_ tension of sterile Universal‐Bac^3^Gel was measured using a Clark‐type O_2_ sensor (OX‐25; Unisense, Aarhus N, Denmark), connected to the Unisense microsensor multimeter S/N 8678 (Unisense, Denmark), a high sensitivity pico‐ampere four‐channel amplifier. Before each measurement, the reference anode and the guard cathode were polarized overnight and calibrated with either water saturated with air or with an anoxic solution of 2% (w/w) sodium hydrosulfite. Once the calibration was carefully made, a low‐melting‐point agarose consisting of 2% (w/v) agarose in 0.071% (w/v) NaCl (same concentration as in Universal‐Bac^3^Gel) was cast into a Petri dish. Universal‐Bac^3^Gel were placed over the agarose layer. Microsensors with a tip diameter of 50 µm (OX‐50) were positioned at the air‐Bac^3^Gelinterface (“depth zero”) using a motorized micromanipulator (Unisense, Denmark). Measurements were performed at the center of the hydrogels starting at their surface (0 mm) through their thickness, every 100 µm in triplicate, until the tip completely penetrated the whole structure. The maximum depth reached by the tip, termed end depth, was 3000 µm. The Unisense software SensorTrace automatically converts the signal from partial pressure (O_2_ tension) to the equivalent O_2_ concentration in µmol L^−1^.

### 2.4 Bacterial Strains and Culture Conditions

The microorganisms used were *Enterococcus faecium* (*E. faecium*), *Staphylococcus aureus* ATCC 25923 (*S. aureus*), *Pseudomonas aeruginosa* PAO1 (*P. aeruginosa*), and *Enterobacter cloacae* (*E. cloacae*) kindly supplied by Prof. Livia Visai (Department of Molecular Medicine, University of Pavia, Italy) and stored at −80°C; *Klebsiella pneumoniae* ATCC 13883 (*K. pneumoniae*), *Acinetobacter baumannii* LMG 1025 (*A. baumannii*) were kindly provided by Doctor Ângelo Filipe Santos Luis (Health Sciences Research Center, Beira Interior University) and stored at −80°C. Bacteria were grown in their appropriate medium overnight, under aerobic conditions, at 37°C using a shaker incubator (VDRL Stirrer 711/CT, Asal S.r.l., Milan, Italy). Brain Heart Infusion (BHI, Sigma‐Aldrich, 53286, Lot#BCCD5575) was used for *K. pneumoniae*, *P. aeruginosa*, *S. aureus*, *E. faecium* and *E. cloacae*. Mueller Hinton (MH, Sigma‐Aldrich, 70192, Lot# BCCC5707) was used to grow *A. baumannii* LMG 1025. The inocula were diluted to a final concentration of 1 × 10^4^ bacteria/mL as determined by comparing the optical density (OD_600_) of the sample with a standard curve relating OD_600_ to cell number (Restivo et al. 2024). Studies focused on the ESKAPE panel of six nosocomial pathogens (which exhibit multidrug resistance and high virulence (Mulani et al. 2019) given their priority status in new antibiotic development efforts. To evaluate the growth in both conditions, 100 µL of bacterial suspension was incubated overnight in Universal‐Bac^3^Gel, at 37°C. Inoculations were carried out for 24 and 48 h. Planktonic cultures were conducted as controls of the experiment, while sterile BHI, MH, and Luria‐Bertani broth, as well as sterile Universal‐Bac^3^Gel were used as controls of sterility. Prior to each experiment, bacterial purity was verified by plating on selective agar. All experiments were performed as biological triplicates (*n* = 3 independent experiments conducted on separate days), each including a technical duplicate.

### 2.5 Spot Plating and Colony‐Forming Unit (CFU) Counting

The spot plating method was selected as a substitute for the conventional plating method to avoid time‐consuming steps, reduce material costs, and obtain high‐throughput data (Zeden and Gründling 2023). After 24 and 48 h of colonization, bacteria grown in Universal‐Bac^3^Gel were determined through CFU plating. Before performing the dilutions, the Universal‐Bac^3^Gel was dissolved using Bac^3^Gel dissolution medium (50 mM sodium citrate) to retrieve bacteria grown within the hydrogel structure. Serial dilutions were performed using 96‐well culture plates, with each well containing 90 µL of 0.9% NaCl, to which 10 µL of the bacterial suspension was added. After thorough pipetting in each well, several dilutions were made for the same starting sample. Using a calibrated micropipette, 10 μl aliquots from the selected four dilutions were applied on top of the dedicated agar plate. The plates were left in the laminar airflow chamber for the droplets to dry off (approx. 8–10 min), after which they were incubated overnight at 37°C to allow colony formation. Planktonic cultures were conducted as controls. For this, the same steps were followed, except for the dissolving agent. For the sterility controls, 10 µL of culture medium was taken from the 96‐well plate used in the experiment and directly spotted onto an agar plate, as well as 10 µL of dissolved Universal‐Bac^3^Gel. Each experiment was performed in triplicate at two different times. After overnight incubation, CFU counting was performed manually by counting the individual colonies (dots) present on the agar plate.

### 2.6 Confocal Laser Scanning Microscope Analysis

Bacteria were grown overnight in Universal‐Bac^3^Gel as previously described. After 24 and 48 h of inoculation in Universal‐Bac^3^Gel, the supernatant was carefully removed, and the hydrogels were washed twice with phosphate‐buffered saline (PBS). Bacterial viability in Universal‐Bac^3^Gel was assessed using the LIVE/DEAD BacLight Viability Kit (Molecular Probes, Eugene, OR, USA). For staining, SYTO9 was incubated for 10 min, followed by PI for 30 s. Bacteria were then observed using a Leica TCS SP8 DLS Confocal Laser Scanning Microscope (CLSM) (Leica, Wetzlar, Germany). Viable bacteria were visualized with a 25× multi‐immersion phase contrast objective. The orthogonal projections and 3D reconstructions were acquired from different (*n* = 5) regions of interest. Scale bars were generated using the LAS X software. The fluorescence intensity of the red and green channels of the 5 acquired images was evaluated using FIJI software. (Schindelin et al. 2012). This qualitative imaging complements the quantitative CFU data, providing insight into the 3D organization and viability of the bacterial communities inside the hydrogel.

### 2.7 Ciprofloxacin Susceptibility Assay

Minimum inhibitory concentrations (MICs) of ciprofloxacin for each bacterial strain were determined by broth microdilution according to EUCAST guidelines. Following incubation at 37°C for 24 h, MIC values were defined as the lowest ciprofloxacin concentration that completely inhibited visible bacterial growth. The MIC values obtained were 2, 0.125, 32, 0.25, 1 and 1 µg mL^−1^ for *A. baumannii, E. cloacae, E. faecium, K. pneumoniae, P. aeruginosa and S. aureus*, respectively.

To evaluate antimicrobial susceptibility under planktonic and biofilm‐relevant conditions, bacterial cultures were grown either in liquid medium (planktonic cultures) or within Universal‐Bac3Gel for 24 h at 37°C. Ciprofloxacin was subsequently added at a concentration corresponding to 10× the MIC determined for each strain in order to assess biofilm‐associated antimicrobial tolerance. Untreated cultures receiving fresh medium alone were included as controls. Following an additional 24 h incubation period, bacterial viability was quantified by colony‐forming unit (CFU) enumeration as described above. Briefly, bacteria were recovered from planktonic cultures or dissolved Universal‐Bac3Gel, serially diluted in sterile saline solution, and plated on the appropriate agar media. Colonies were counted after overnight incubation at 37°C and results were expressed as CFU mL^−1^. All experiments were performed in triplicate.

### 2.8 Crystal Violet Assay

Biofilm‐associated biomass accumulation within Universal‐Bac3Gel was assessed by crystal violet staining, adapted from the microtiter plate biofilm assay described by O'Toole (2011). Briefly, bacterial suspensions were inoculated either into Universal‐Bac3Gel or into conventional tissue culture plate (TCP) 96‐well plates and incubated at 37°C for 24 h. Following incubation, culture supernatants were carefully removed, and hydrogels or plastic wells were gently washed twice with PBS to eliminate non‐associated cells. A 0.1% (w/v) crystal violet solution was then added to each well and incubated for 15 min at room temperature. Excess stain was removed by washing with distilled water, and the retained dye was solubilized using 30% acetic acid. Absorbance was measured at 590 nm using a microplate reader. Wells containing sterile Universal‐Bac3Gel were included as controls, and their absorbance values were subtracted from sample readings to obtain background‐corrected values. All experiments were performed in triplicate.

### 2.9 Statistical Analysis

All statistical analyses were performed using GraphPad Prism 9 (GraphPad Inc., San Diego, CA, USA). Data are presented as mean ± standard deviation (SD) of three independent biological replicates (*n* = 3). Each biological replicate was performed on a separate day and included technical duplicates. CFU values are reported as the mean of the corresponding technical replicates. Time‐course growth experiments and ciprofloxacin susceptibility assays were analyzed using ordinary two‐way ANOVA followed by Bonferroni's multiple‐comparisons post hoc test. Differences in biofilm‐associated biomass quantified by crystal violet staining were assessed using an unpaired two‐tailed Student's *t*‐test to compare plastic and Universal‐Bac^3^Gel culture conditions for each bacterial strain. Statistical significance was defined as follows: *p < 0.05, **p < 0.01, ***p < 0.001, and ****p < 0.0001. Values of *p* > 0.05 were considered not statistically significant (ns).

## 3 Results

### 3.1 Characteristics of Universal‐Bac^3^Gel

**Figure 2:**
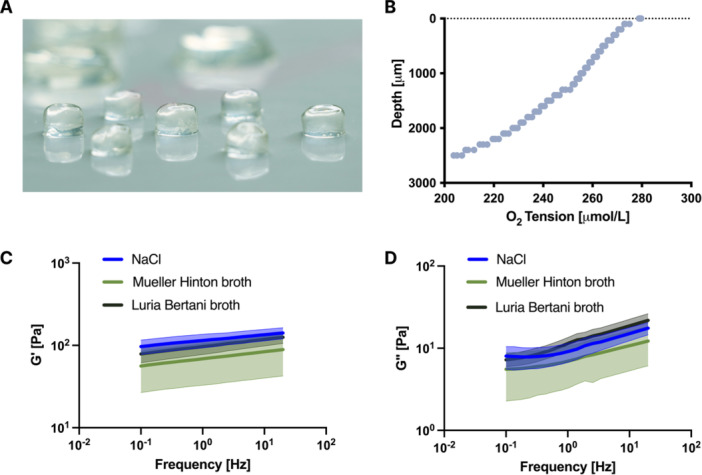
Characterization of Universal‐Bac^3^Gel as a biofilm‐relevant matrix. (A) Macroscopic image depicting the cylindrical‐shape of Bac^3^Gel; (B) Heterogeneous distribution of oxygen tension along Bac^3^Gel depth displaying a diffusion‐driven gradient; (C, D) Frequency‐dependent storage (G′) and loss (G″) moduli measured for gels hydrated with NaCl, Mueller‐Hinton, or Luria‐Bertani media. The area around the lines in C and D represents the standard deviation.

The hydrogel is a transparent, self‐supporting cylindrical matrix compatible with standard multiwell formats (Figure 2A). Oxygen profiling along the gel depth revealed a stable gradient from approximately 290 µmol L^−1^ at the surface to ~195 µmol L^−1^ near 3 mm depth (Figure 2B). Rheological characterization demonstrated that Universal‐Bac^3^Gel possesses viscoelastic properties consistent with extracellular polymeric substance (EPS)‐rich matrices with a storage modulus (G′) higher than the loss modulus (G″) (Figure 2C,D), indicating solid‐*like* behavior essential for maintaining biofilm structure under shear or mechanical stress. The moduli remained stable across gels hydrated with NaCl, Mueller‐Hinton, or Luria‐Bertani media, confirming mechanical robustness and reproducibility under standard microbial culture conditions. Oxygen tension measurements were conducted in sterile Universal‐Bac3Gel to characterize the baseline physicochemical properties of the matrix.

### 3.2 Colonization and Biofilm Formation by Gram‐Positive Eskape Pathogens in Universal‐Bac^3^Gel

**Figure 3:**
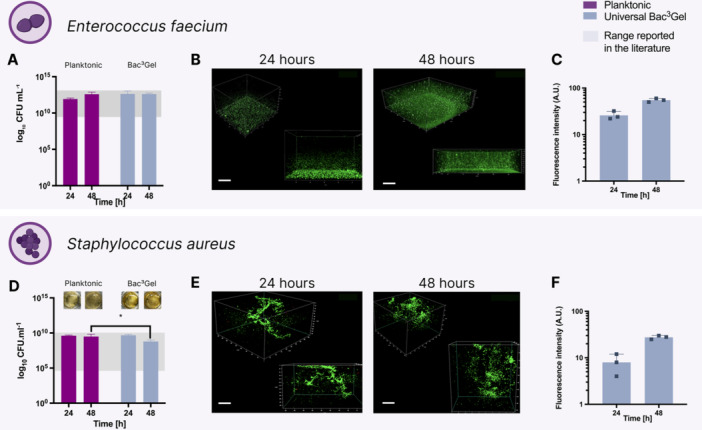
Growth and biofilm formation of Gram‐positive ESKAPE pathogens in Universal‐Bac^3^Gel. (A, D) Viable bacterial counts (log10 CFU mL^−1^) of E. faecium and S. aureus cultured under planktonic (purple) and Universal‐Bac^3^Gel (light blue) conditions for 24 h and 48 h at 37°C. The shaded gray area indicates the CFU range reported in infected mucosal environments. Data are presented as mean ± SD (*n* = 3). Statistical significance is indicated in the figure (*p < 0.05). (B, E) Representative three‐dimensional CLSM reconstructions of viable bacterial populations within Universal‐Bac^3^Gel at 24 h and 48 h. Scale bar = 50 µm. (C, F) Quantification of green fluorescence intensity from CLSM image stacks, corresponding to viable biofilm‐associated biomass within the hydrogel. Data are presented as mean ± SD (*n* = 3).

The two Gram‐positive bacteria, *Enterococcus faecium* (Kim et al. 2021) and *Staphylococcus aureus* (Olsen et al. 2013), are major contributors to hospital‐acquired infections and are known for their ability to form robust and persistent biofilms. To investigate the colonization potential of Gram‐positive ESKAPE pathogens in a 3D biofilm‐relevant environment, both strains were inoculated on top of Universal‐Bac^3^Gel, and their permeation and colonization were monitored over 24 and 48 h. As shown in the CFU quantification, both *E. faecium* (Figure 3A) and *S. aureus* (Figure 3D) demonstrated high viability within the hydrogel, reaching viable counts ranging from approximately 8 to 12 log_10_ (CFU mL^−1^) across both time points. These levels were statistically equivalent (*p* > 0.05) to planktonic culture controls (purple bars), indicating that the matrix does not hinder bacterial growth and instead supports sustained proliferation over time.

Microscopy‐based qualitative analysis revealed distinct 3D spatial organization for the two Gram‐positive strains (Figure 3B,E). *E. faecium* exhibited a relatively homogeneous distribution of green fluorescence throughout Bac^3^Gel, forming a dense but dispersed biofilm (Figure 3B). The structure appeared compact, without large aggregates, suggesting a uniform colonization pattern reminiscent of a lawn‐like biofilm. *S. aureus*, in contrast, displayed a markedly different architecture, forming dense and spatially localized aggregates typical of microcolony‐based biofilm structures (Figure 3E). These clusters are consistent with the known ability of *S. aureus* to form mature and structured biofilms with high biomass density (Schilcher and Horswill 2020).

Live/dead staining indicated predominantly live (green) cells in both cases, with only sparse dead (red) cells visible, suggesting the bacteria remained highly viable in the matrix. Quantification of fluorescence intensity in 3D reconstructions (Figure 3C,F) provided a measure of biofilm biomass. *E. faecium* (Figure 3C) displayed significantly brighter and more localized fluorescent regions, indicating higher biofilm density and biomass accumulation. In contrast, *S. aureus* (Figure 3F) showed a more moderate fluorescent signal that was more heterogeneously distributed, consistent with its fewer but larger clumps.

**Figure 4:**
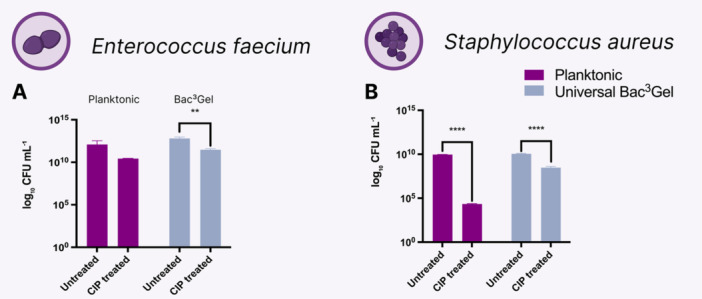
Effect of ciprofloxacin treatment on Gram‐positive ESKAPE pathogens cultured under planktonic and Universal‐Bac^3^Gel conditions. Viable bacterial counts (log10 CFU mL^−1^) of E. faecium (A) and S. aureus (B) cultured under planktonic (purple) or Universal‐Bac^3^Gel (light blue) conditions at 37°C. After 24 h of growth, cultures were either left untreated or exposed to ciprofloxacin (CIP) at 10 × the strain‐specific MIC for an additional 24 h. Data are presented as mean ± SD of three independent biological replicates (*n* = 3). Statistical analysis was performed using two‐way ANOVA followed by Bonferroni's multiple‐comparisons test. Statistical significance is indicated in the figure (**p < 0.01, ****p < 0.0001).

To further evaluate whether bacterial growth within Universal‐Bac^3^Gel was associated with altered antimicrobial susceptibility, ciprofloxacin tolerance was assessed using strain‐specific concentrations corresponding to 10 × MIC (Figure 4). In both species, ciprofloxacin treatment reduced viable bacterial counts compared with untreated controls. For *E. faecium*, the reduction in viability was significantly less pronounced in Universal‐Bac^3^Gel than under planktonic conditions, resulting in significantly higher CFU counts following treatment (**p < 0.01). A similar trend was observed for *S. aureus*, where bacterial survival remained substantially higher in Universal‐Bac^3^Gel than in planktonic cultures after ciprofloxacin exposure (****p < 0.0001). These results indicate that bacterial growth within the hydrogel is associated with reduced susceptibility to ciprofloxacin.

**Figure 5:**
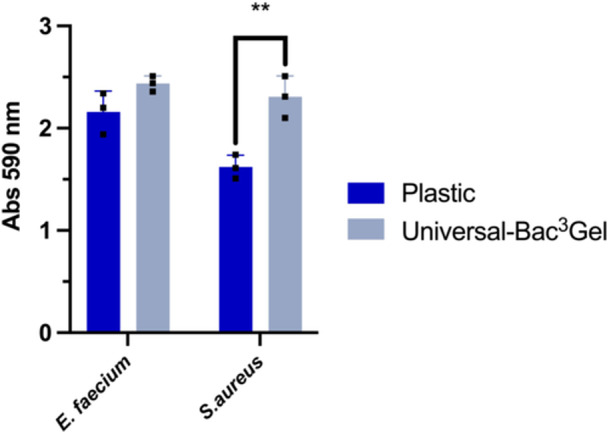
Biofilm‐associated biomass accumulation of Gram‐positive ESKAPE pathogens cultured in Universal‐Bac^3^Gel. Quantification of biofilm‐associated biomass by crystal violet staining for E. faecium and S. aureus after 24 h of incubation at 37°C under conventional surface‐associated culture conditions (plastic) or within Universal‐Bac^3^Gel. Biofilm biomass was assessed by measuring crystal violet absorbance at 590 nm. Background‐corrected absorbance values were obtained by subtracting the mean absorbance of gel‐only controls. Data are presented as mean ± SD of three independent biological replicates (*n* = 3). Statistical analysis was performed using Student's *t*‐test to compare biofilm biomass between conventional surface‐associated culture conditions and Universal‐Bac^3^Gel for each strain. Statistical significance is indicated in the figure (**p < 0.01).

Biofilm‐associated biomass accumulation was further quantified by crystal violet staining after 24 h of incubation (Figure 5). Both Gram‐positive pathogens produced substantial biomass under conventional surface‐associated culture conditions and within Universal‐Bac^3^Gel. While *E. faecium* displayed comparable absorbance values in both systems, *S. aureus* exhibited significantly higher crystal violet staining in Universal‐Bac^3^Gel than under conventional culture conditions (*p* < 0.01), indicating increased biomass accumulation within the three‐dimensional matrix.

### 3.3 Colonization and Biofilm Formation by Gram‐Negative Eskape Pathogens in Universal‐Bac3gel

**Figure 6:**
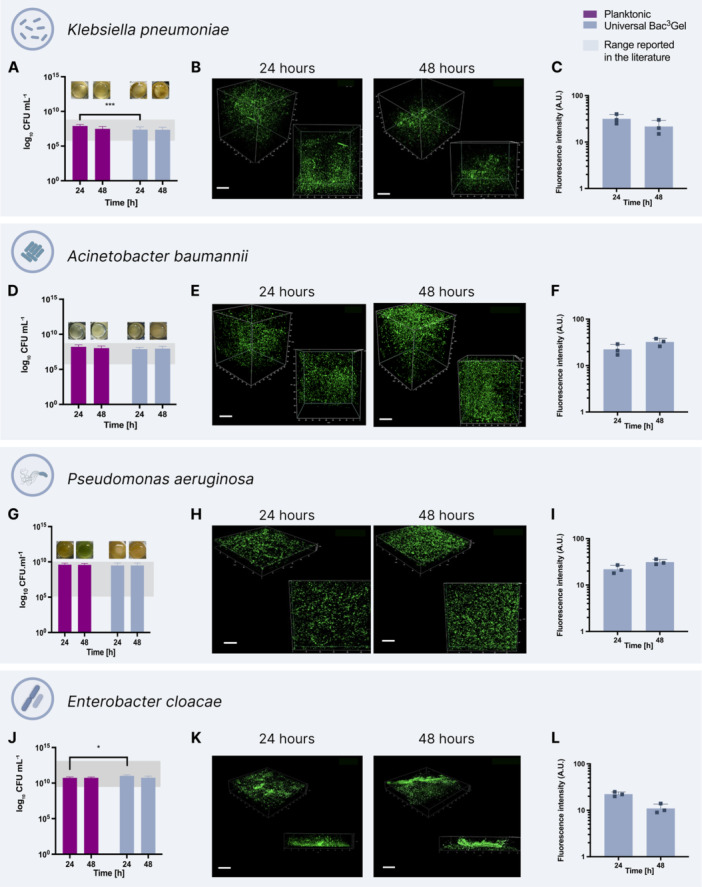
Growth and biofilm formation of Gram‐negative ESKAPE pathogens in Universal‐Bac^3^Gel. (A, D, G, J) Viable bacterial counts (log10 CFU mL^−1^) of K. pneumoniae, A. baumannii, *P. aeruginosa* and *E. cloacae* cultured under planktonic (purple) and Universal‐Bac^3^Gel (light blue) conditions for 24 and 48 h at 37°C. The shaded gray area indicates the CFU range reported in infected mucosal environments. Data are presented as mean ± SD (*n* = 3). Statistical significance is indicated in the figure. (B, E, H, K) Representative three‐dimensional CLSM reconstructions of viable bacterial populations within Universal‐Bac^3^Gel at 24 and 48 h. Scale bar = 50 µm. (C, F, I, L) Quantification of green fluorescence intensity from CLSM image stacks, corresponding to viable biofilm‐associated biomass within the hydrogel. Data are presented as mean ± SD (*n* = 3).

The Gram‐negative ESKAPE pathogens *Klebsiella pneumoniae*, *Acinetobacter baumannii*, *Pseudomonas aeruginosa*, and *Enterobacter cloacae* also represent critical agents of nosocomial infections and are characterized by their intrinsic and acquired resistance mechanisms, as well as their capacity to form complex biofilm communities (Santajit and Indrawattana 2016). We evaluated their colonization potential in the 3D biofilm‐relevant environment Universal‐Bac^3^Gel with cultures at 24 and 48 h. CFU analysis revealed that all strains remained viable within the hydrogel, maintaining viable counts ranging from approximately 6–10 log_10_ (CFU mL^−1^) (Figure 6A,D,G,J), similar to planktonic conditions. In most cases, CFU counts in Universal‐Bac^3^Gel were comparable to planktonic controls. Notably, *K. pneumoniae* and *E. cloacae* showed a slower growth (~1–2 log) in CFU at 24 h in Universal‐Bac^3^Gel relative to planktonic broth (Figure 6A,J), suggesting a transient adaptation phase. By 48 h, however, their counts in Universal‐Bac^3^Gel had increased and were closer to planktonic levels (indicating these strains eventually proliferated well after the initial lag). In contrast, *A. baumannii* and *P. aeruginosa* maintained stable growth without any significant initial deficit. The number of bacteria in Universal‐Bac^3^Gel was similar to their planktonic counterparts at both 24 and 48 h, indicating steady growth with no apparent adaptation lag (Figure 6D,G).

The intensity and distribution of green fluorescence signals in the 3D reconstructions correlate with viable bacterial biomass, supporting the ability of Universal‐Bac^3^Gel to sustain long‐term colonization and species‐specific biofilm architectures in Gram‐negative pathogens. Microscopy‐based qualitative analysis confirmed distinct 3D biofilm organization within Universal‐Bac^3^Gel (Figure 6B,E,H,K). *K. pneumoniae* and *E. cloacae* formed compact yet dispersed aggregates, small clusters of cells scattered throughout the matrix, consistent with their capacity for early biofilm development. *A. baumannii* displayed a more homogeneous fluorescence throughout Universal‐Bac^3^Gel (Figure 6E), suggestive of a diffuse colonization pattern with individual cells or small groups spread rather evenly. No large clusters were observed at 48 h observation after culturing *A. baumannii*. *P. aeruginosa*, by contrast, developed dense and spatially localized microcolonies (Figure 6H)—tightly packed spherical clusters—characteristic of its mature biofilm phenotype.

As with the Gram‐positives, live/dead staining showed that the vast majority of cells in these biofilms were alive (green), with very limited red signals, indicating that Universal‐Bac^3^Gel provides a conducive environment for the growth of these pathogens over 48 h. The intensity and distribution of green fluorescence correlated with viable bacterial biomass (Figure 6C,F,I,L). *P. aeruginosa* had very high localized green intensity (reflecting its large microcolonies), whereas *A. baumannii* showed a lower, evenly distributed signal. *K. pneumoniae* and *E. cloacae* had intermediate patterns. These species‐specific biofilm architectures underscore the importance of using a 3D‐relevant environment, as differences in spatial organization would be flattened or missed entirely in 2D culture.

**Figure 7:**
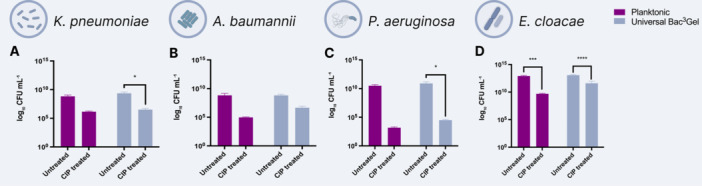
Effect of ciprofloxacin treatment on Gram‐negative ESKAPE pathogens cultured under planktonic and Universal‐Bac^3^Gel conditions. Viable bacterial counts (log10 CFU mL^−1^) of K. pneumoniae (A), A. baumannii (B), P. aeruginosa (C), and E. cloacae (D) cultured under planktonic (purple) or Universal‐Bac^3^Gel (light blue) conditions at 37°C. After 24 h of growth, cultures were either left untreated or exposed to ciprofloxacin (CIP) at 10 × the strain‐specific MIC for an additional 24 h. Data are presented as mean ± SD of three independent biological replicates (*n* = 3). Statistical analysis was performed using two‐way ANOVA followed by Bonferroni's multiple‐comparisons test. Statistical significance is indicated in the figure (*p < 0.05, ***p < 0.001, ****p < 0.0001).

The effect of biofilm growth on antimicrobial susceptibility was assessed using ciprofloxacin at 10× the strain‐specific MIC (Figure 7). Ciprofloxacin treatment reduced bacterial viability in all Gram‐negative species. However, bacteria cultured within Universal‐Bac^3^Gel generally retained higher viable counts than their planktonic counterparts following treatment. Significant differences between planktonic and hydrogel cultures were observed for *K. pneumoniae* (*p < 0.05), *P. aeruginosa* (*p < 0.05), and *E. cloacae* (****p < 0.0001), whereas *A. baumannii* displayed a similar trend that did not reach statistical significance. These findings indicate reduced ciprofloxacin susceptibility in bacteria cultured within the hydrogel environment.

**Figure 8:**
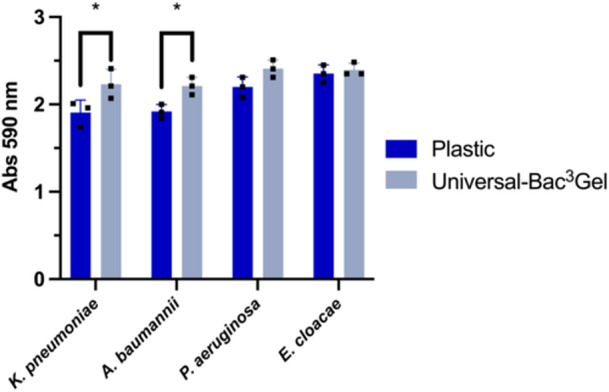
Biofilm‐associated biomass accumulation of Gram‐negative ESKAPE pathogens cultured in Universal‐Bac^3^Gel. Quantification of biofilm‐associated biomass by crystal violet staining for K. pneumoniae, A. baumannii, P. aeruginosa, and E. cloacae after 24 h of incubation at 37°C under conventional surface‐associated culture conditions (plastic) or within Universal‐Bac^3^Gel. Biofilm biomass was assessed by measuring crystal violet absorbance at 590 nm. Background‐corrected absorbance values were obtained by subtracting the mean absorbance of gel‐only controls. Data are presented as mean ± SD of three independent biological replicates (*n* = 3). Statistical analysis was performed using Student's t‐test to compare biofilm biomass between conventional surface‐associated culture conditions and Universal‐Bac^3^Gel for each strain. Statistical significance is indicated in the figure (*p < 0.05).

Crystal violet staining confirmed the accumulation of biofilm‐associated biomass by all Gram‐negative pathogens after 24 h of incubation (Figure 8). Biomass levels were generally similar between the two culture conditions, although significant increases in crystal violet absorbance were observed for *K. pneumoniae* and *A. baumannii* (*p* < 0.05). In contrast, *P. aeruginosa* and *E. cloacae* displayed comparable biomass levels under both conditions. Overall, these results demonstrate that all four Gram‐negative species successfully accumulated measurable biofilm‐associated biomass within Universal‐Bac^3^Gel.

## 4 Discussion

The results presented in this study demonstrate that Universal‐Bac^3^Gel provides a stable, reproducible, and physicochemically relevant 3D microenvironment for modeling biofilm‐associated growth of ESKAPE pathogens (Figure 1). The hydrogel's structural and rheological properties—transparency, self‐supporting nature, and viscoelastic behavior with G′ > G″— closely resemble those of natural EPS‐rich biofilm matrices, whose elastic moduli typically range from 0.2 to 25 Pa depending on species and maturation stage (Klapper et al. 2002; Pavlovsky et al. 2015), which falls within the storage modulus of Universal‐Bac^3^Gel (2–200 Pa) (Figure 2C,D). The presence of a measurable oxygen gradient across the hydrogel depth further supports its ability to reproduce diffusion limitations typical of in vivo mucosal environments (Figure 2B). Together, these features generate a stratified habitat where microorganisms can proliferate and self‐organize into biofilm‐like communities.

A key outcome of this work is that all six ESKAPE pathogens maintained high viability and CFU counts comparable to their planktonic controls, confirming that Universal‐Bac^3^Gel supports bacterial growth. Confocal microscopy revealed pronounced differences in 3D spatial organization between species, showing that biofilm architecture within the hydrogel is both species‐specific and influenced by the viscoelastic environment.

Among the Gram‐positive pathogens, distinct colonization patterns were observed within Universal‐Bac3Gel. *E. faecium* exhibited a homogeneous distribution of viable cells throughout the matrix (Figure 3B) (Kim et al. 2021), whereas *S. aureus* formed spatially localized dense microcolonies (Figure 3E) (Schilcher and Horswill 2020). In Universal‐Bac^3^Gel, *S. aureus* exhibited more compact and localized aggregates throughout its matrix, compared to the previous gradient‐based platform (Pacheco et al. 2023), possibly associated with the lack of mucin and the resulting reduction of biochemical adhesion cues. For the Gram‐negative species, distinct colonization patterns were also evident. *K. pneumoniae* and *E. cloacae* initially displayed slower growth at 24 h but by 48 h both reached CFU levels comparable to planktonic cultures (Figure 6A‐J). *A. baumannii* (Figure 6E) displayed a diffuse and homogeneous colonization pattern throughout the matrix (Martí et al. 2011). *P. aeruginosa* (Figure 6H) formed dense spherical microcolonies characteristic of highly structured biofilm communities (Moradali et al. 2017). Similarly to *S. aureus, P. aeruginosa* in Universal‐Bac^3^Gel exhibited a more homogeneous distribution pattern than in the previous gradient‐based platform (Pacheco et al. 2023). This outcome further supports that mucin impacts bacterial spatial organization.

A comparative overview of the species‐specific growth kinetics and three‐dimensional architectures observed in Universal‐Bac3Gel is provided in Table 1.

**Table 1:** Summary of ESKAPE pathogen behavior in universal‐Bac3Gel.

Pathogen	Growth Kinetics	3D architecture	Biofilm‐associated features
*E. faecium*	High viability at 24‐48 h; comparable to planktonic cultures	Dense, homogeneous lawn‐like distribution	Diffuse biofilm architecture; extensive matrix colonization
*S. aureus*	High viability at 24‐48 h; comparable to planktonic cultures	Localized dense microcolonies	Clustered biofilm organization
*K. pneumoniae*	Initial lag at 24 h; recovery by 48 h	Compact dispersed aggregates	Delayed adaptation to the 3D environment
*A. baumannii*	Stable growth at 24‐48 h	Homogeneous diffuse colonization	Uniform distribution throughout the matrix
*P. aeruginosa*	Stable growth at 24‐48 h	Dense spherical microcolonies	Highly localized biofilm structures
*E. cloacae*	Initial lag at 24 h; recovery by 48 h	Compact dispersed aggregates	Adaptation pattern similar to *K. pneumoniae*

The diversity of architecture observed across species underscores how differences in bacterial physiology, extracellular matrix composition, and motility interact with the mechanical properties of the surrounding environment to determine biofilm morphology. Filamentous or clustered assemblies within Universal‐Bac^3^Gel are consistent with recent physical models showing that cell shape and mechanical confinement regulate biofilm self‐organization (Charlton et al. 2025). In such environments, filamentation lowers the percolation threshold and promotes the formation of connected networks through cluster‐cluster aggregation and mechanical feedback. The viscoelastic properties of Universal‐Bac^3^Gel likely provide similar mechanical cues: stiffness gradients, diffusion limitations, and stress relaxation collectively guide the emergence of anisotropic and filamentous growth, stabilizing biofilm structures over time. As highlighted by Savorana et al. (2025) the balance between storage and loss moduli (G′ > G″) determines the extent of mechanical coupling between cells and their surroundings, influencing biofilm cohesion, deformation, and recovery. The elastic modulus of Universal‐Bac^3^Gel therefore not only supports physical stability but also promotes biologically meaningful organization and persistence.

In addition to supporting bacterial growth and spatial organization, Universal‐Bac^3^Gel promoted the development of functional biofilm‐associated phenotypes. Beyond the species‐specific architectures observed by CLSM, bacteria cultured within the hydrogel exhibited reduced susceptibility to ciprofloxacin and accumulated substantial biofilm‐associated biomass, indicating that the matrix supports both structural and functional characteristics commonly associated with biofilm growth.

From a biological perspective, structured three‐dimensional microbial communities are frequently associated with increased tolerance to antimicrobial agents, metabolic cooperation, and protection from environmental stresses (Pabst et al. 2016). To investigate whether bacteria cultured within Universal‐Bac^3^Gel exhibit functional traits consistent with biofilm growth, we evaluated susceptibility to ciprofloxacin, a broad‐spectrum fluoroquinolone active against both Gram‐positive and Gram‐negative pathogens (Hooper 2000) (Figures 4 and 7).

Ciprofloxacin susceptibility assays revealed that bacteria cultured within Universal‐Bac^3^Gel generally retained higher viability following antibiotic exposure than their planktonic counterparts (Figures 4 and 7). This trend was observed across both Gram‐positive and Gram‐negative ESKAPE pathogens despite the use of ciprofloxacin at concentrations corresponding to 10× the strain‐specific MIC. As ciprofloxacin exerts its antimicrobial activity through inhibition of DNA gyrase and topoisomerase IV (Drlica et al. 1997), the reduced susceptibility observed within Universal‐Bac^3^Gel is unlikely to reflect intrinsic resistance alone and instead suggests that the three‐dimensional environment promotes biofilm‐associated tolerance mechanisms. Several factors may contribute to this phenotype, including diffusion limitations within the hydrogel matrix, the formation of oxygen and nutrient gradients, and the presence of metabolically heterogeneous bacterial populations that are less susceptible to antibiotic‐mediated killing (Stewart and Franklin 2008; Hall and Mah 2017).

Crystal violet staining provided additional evidence supporting biofilm establishment within Universal‐Bac^3^Gel® (Figures 5 and 8). All tested ESKAPE pathogens accumulated substantial biofilm‐associated biomass within the hydrogel, with several species exhibiting significantly greater biomass than under conventional surface‐associated culture conditions. These results complement the CLSM observations (Figures 3 and 6) and provide an independent quantitative assessment of biofilm‐associated biomass formation.

The applicability of Bac3Gel‐derived biomimetic matrices for biofilm research is further supported by recent independent work demonstrating the formation of mature mono‐, dual‐, and poly‐species biofilms and the evaluation of phage‐ and antibiotic‐based therapies in a cystic fibrosis mucus model (Glonti et al. 2026).

Taken together, the CLSM analyses, ciprofloxacin susceptibility assays and crystal violet staining results provide complementary structural and functional evidence that Universal‐Bac^3^Gel supports the development of biofilm‐associated phenotypes beyond simple bacterial proliferation.

## 5 Conclusions

This study demonstrates that all six ESKAPE priority pathogens can successfully colonize, proliferate, and establish biofilm‐associated communities within Universal‐Bac3Gel, a three‐dimensional biomimetic hydrogel that reproduces key structural and physicochemical features of biofilm environments, including diffusion gradients and viscoelastic confinement. These findings validate Universal‐Bac3Gel as a growth‐compatible platform for the entire ESKAPE pathogen panel.

Confocal microscopy revealed species‐specific three‐dimensional architectures within the matrix, ranging from diffuse colonization patterns to dense microcolony formation. Furthermore, ciprofloxacin susceptibility assays demonstrated reduced susceptibility of bacteria cultured within Universal‐Bac3Gel compared with their planktonic counterparts, while crystal violet staining confirmed the accumulation of biofilm‐associated biomass. Together, these results indicate that the platform supports not only bacterial growth and spatial organization but also functional phenotypes commonly associated with biofilm development.

While additional studies incorporating multidrug‐resistant clinical isolates, standardized quantitative biofilm analyses, and benchmarking against established biofilm models will further expand the characterization of the platform, the present findings provide complementary structural and functional evidence supporting the use of Universal‐Bac3Gel as a reproducible three‐dimensional model for investigating ESKAPE pathogen colonization, biofilm formation, and antimicrobial susceptibility under controlled in vitro conditions.

## Author Contributions

**Emanuela Peluso:** data curation (lead), formal analysis (lead), investigation (lead), methodology (equal), visualization (lead), writing – review and editing (lead). **Sebastião van Uden:** conceptualization (lead), funding acquisition (lead), methodology (lead), project administration (supporting), resources (lead), validation (supporting). **Sonja Visentin:** conceptualization (supporting), funding acquisition (equal), project administration (supporting), supervision (supporting), validation (supporting). **Paola Petrini:** conceptualization (lead), funding acquisition (equal), project administration (supporting), supervision (supporting), validation (supporting). **Daniela Peneda Pacheco:** conceptualization (lead), funding acquisition (lead), methodology (lead), project administration (supporting), resources (lead), supervision (lead), validation (lead). **Livia Visai:** conceptualization (lead), funding acquisition (lead), project administration (lead), resources (lead) supervision (lead), validation (lead).

## Ethics Statement

The authors have nothing to report.

## Conflicts of Interest

D.P.P., S.v.U., S.V., P.P. and L.V. are co‐inventors of the patented technology IT102018000020242A “Three‐dimensional substrate for microbial cultures”. D.P.P. is co‐founder, shareholder, and CTO of Bac^3^Gel, Lda. S.v.U. is co‐founder, shareholder, and CEO of Bac^3^Gel, Lda. S.V., P.P. and L.V. are co‐founders, shareholders, and scientific advisors of Bac^3^Gel, Lda. Bac3Gel is now a registered trademark. The other authors declare no conflicts of interest.

## Data Availability

The data that support the findings of this study are available on request from the corresponding author. The data are not publicly available due to privacy or ethical restrictions.

## Appendix 1 Additional Material

Link A1. Detailed protocol for Universal‐Bac^3^Gel® assays.

Available at: https://drive.google.com/file/d/1Ln8UBYB1B8Pyxd_dd1LfK6v84iNwSX9v/view.
